# Regulation of Heme Synthesis by Mitochondrial Homeostasis Proteins

**DOI:** 10.3389/fcell.2022.895521

**Published:** 2022-06-27

**Authors:** Yvette Y. Yien, Mark Perfetto

**Affiliations:** Department of Medicine and Pittsburgh Heart, Lung and Blood Vascular Medicine Institute, University of Pittsburgh, Pittsburgh, PA, United States

**Keywords:** heme, mitochondria, housekeeping protein, erythroid, yeast, model organisms, mice, zebrafish

## Abstract

Heme plays a central role in diverse, life-essential processes that range from ubiquitous, housekeeping pathways such as respiration, to highly cell-specific ones such as oxygen transport by hemoglobin. The regulation of heme synthesis and its utilization is highly regulated and cell-specific. In this review, we have attempted to describe how the heme synthesis machinery is regulated by mitochondrial homeostasis as a means of coupling heme synthesis to its utilization and to the metabolic requirements of the cell. We have focused on discussing the regulation of mitochondrial heme synthesis enzymes by housekeeping proteins, transport of heme intermediates, and regulation of heme synthesis by macromolecular complex formation and mitochondrial metabolism. Recently discovered mechanisms are discussed in the context of the model organisms in which they were identified, while more established work is discussed in light of technological advancements.

## 1 Introduction

Heme is an amphipathic prosthetic group consisting of a central iron chelated by porphyrin ring and is essential for the function of proteins that regulate a diverse range life-essential redox processes such as oxygen transport, respiration, detoxification, gas sensing, circadian rhythm and control of gene expression (Chen and Paw, 2012; Feng and Lazar, 2012; Girvan and Munro, 2013; Shimizu et al., 2015; Shimizu et al., 2019). Labile heme also functions as a signaling molecule whose binding and dissociation from proteins alters their function (Sun et al., 2004; Suzuki et al., 2004; Tahara et al., 2004; Zenke-Kawasaki et al., 2007; Kobayashi et al., 2017; Martinez-Guzman et al., 2020; Chambers et al., 2021). The redox activity of heme renders it essential in all cell types in microbes and animals. However, this same redox ability and its ability to intercalate into membranes also makes heme potentially cytotoxic as it can catalyze reactions that are deleterious to the cell. Heme intermediates have the same chemical properties. In addition, they are photoactive and cause tissue damage when they accumulate during heme dysregulation. These properties of heme and its intermediates require the tight coupling of heme synthesis to its utilization. While heme is required for all cells of the body, the role of heme, and the amount of heme required is unique to each cell type. Hence, cells have specific regulatory mechanisms that couple heme synthesis with utilization. This is observed in the diversity of phenotypes caused by disorders of heme synthesis, or in loss of function in model organisms (Balwani and Desnick, 2012; Chen et al., 2019; Peoc’h et al., 2019; Poli et al., 2021; Chung et al., 2014; Hildick-Smith et al., 2013; Phillips et al., 2019; Wang et al., 2011; Yien et al., 2017; Troadec et al., 2011; Ducamp et al., 2021). Although defects in heme synthesis are often associated with hematologic diseases, loss of function studies and disease phenotypes make clear that many tissues and pathways depend on the correct regulation of heme synthesis for their function.

While many disorders of heme synthesis are caused by mutations in genes directly associated with heme biosynthesis, iron-sulfur cluster formation or iron transport, a smaller subset of tissue-specific diseases are caused by mutations in genes more often associated with regulation of mitochondrial homeostasis. This latter subset of diseases is less well understood as little is known about the interaction between the mitochondrial homeostatic machinery and heme/iron metabolism. This review hence emphasizes regulation of heme synthesis in metazoans and focuses on tissue specific regulatory mechanisms that enable cells to couple heme synthesis to cellular utilization. In recent years, it has become evident that these regulatory mechanisms have converged into several main categories: 1) Regulation of substrate and cofactor transport; 2) Regulation of protein-protein interactions between the core components of the heme synthesis machinery and other regulatory proteins, and 3) Metabolic regulation of heme synthesis. The first and last steps of heme synthesis takes place in the mitochondria, enabling extensive regulation of heme synthesis rates by control of transport mechanisms. Mitochondria are also the organelles that integrate molecular synthesis with energy metabolism. How metabolism and synthesis of molecules (such as heme) are integrated and co-regulated is poorly characterized, but is likely to play a major role in cell-specific regulation. To shed light on these interfaces, we have chosen to focus this review on how heme synthesis is regulated by mitochondrial physiology.

**FIGURE 1:**
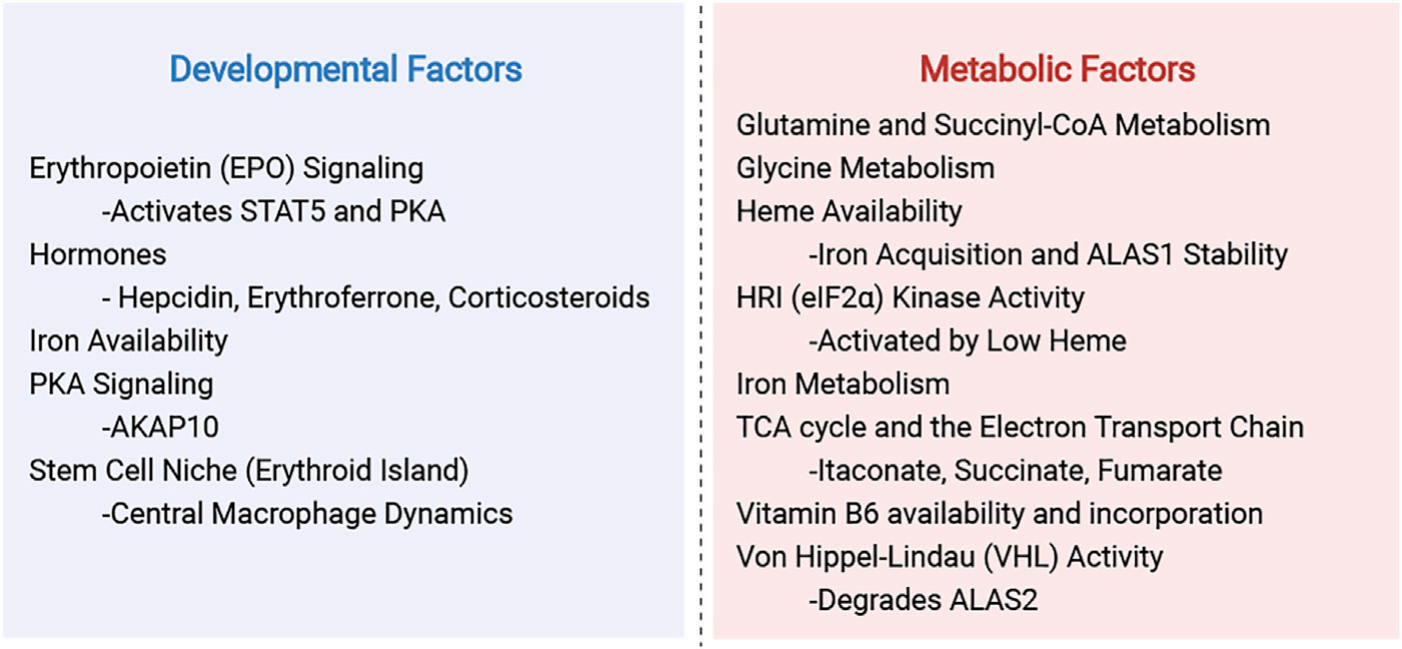
Summary of developmental and metabolic factors that regulate heme synthesis.

One aspect we aim to highlight is the use of diverse model organisms and experimental systems. Many of the fundamental discoveries in eukaryotic heme regulation were made from studies of *S. cerevisiae* yeast and mammalian cell culture, and these continue to be important models in understanding heme biochemistry and genetics. However, cultured yeast and mammalian cells are uniformly bathed in media and cannot recapitulate the metabolic heterogeneity of cell types in multicellular organisms. Hence, our use of vertebrate organisms is key for understanding the role of heme synthesis in tissue development and physiology. We will draw attention to the experimental models and to the strengths and limitations of each experimental system when interpreting their findings. This is important for understanding the complexity of heme regulation in the context of mitochondrial function, which adapts to the biology of different cell types in their metabolic or developmental niches (summarized in Figure 1).

## 2 Heme Synthesis

**FIGURE 2:**
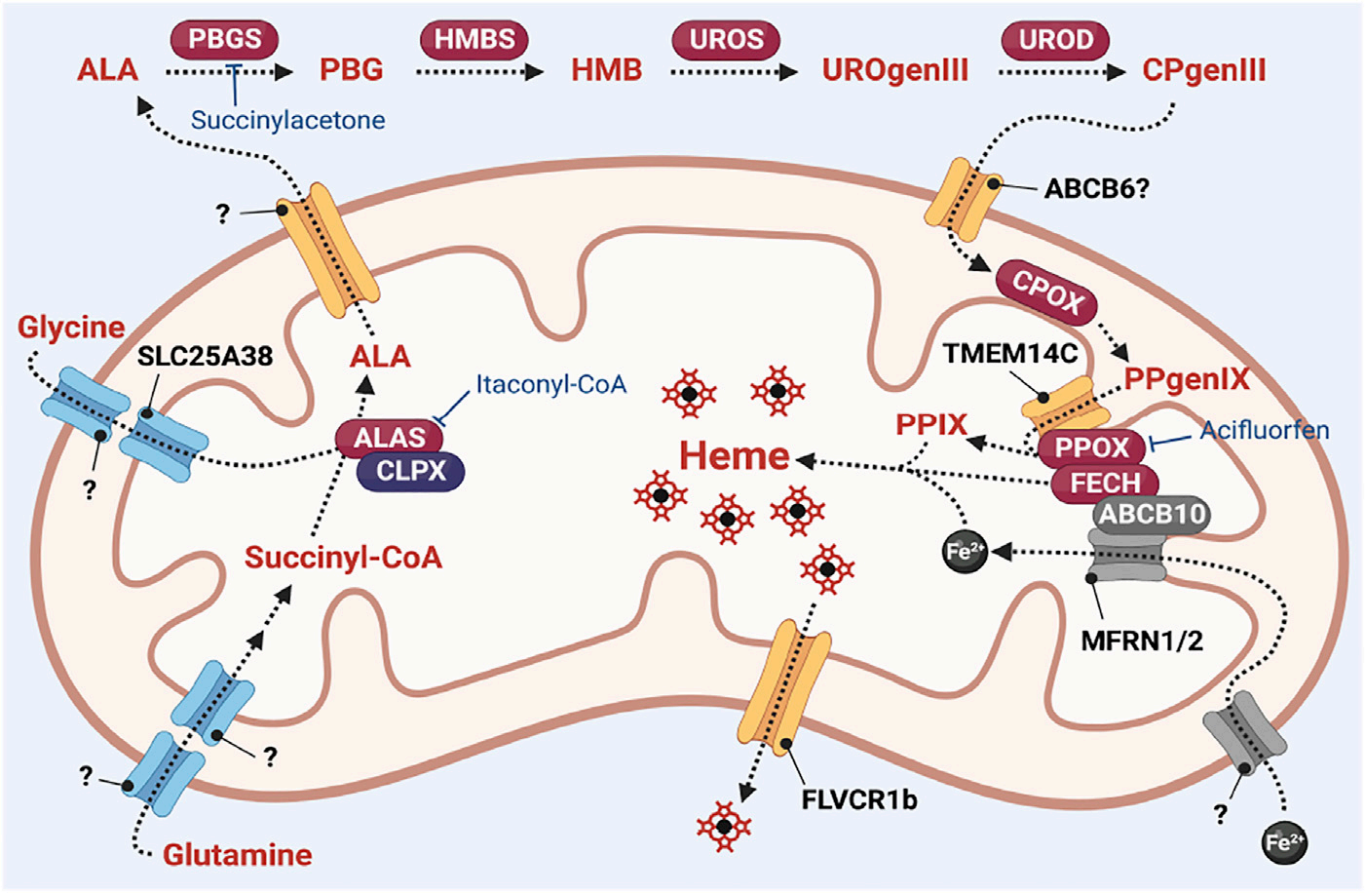
Overview of heme synthesis. Heme synthesis requires the activity of its pathway enzymes (indicated in red ovals). The activities of these enzymes are regulated by interaction with mitochondrial homeostasis proteins such as CLPX and ABCB10. Heme synthesis is also regulated by transport proteins such as TMEM14C, and transport of iron *via* the MFRN proteins. Many transporters of heme intermediates have yet to be identified. Pharmacological inhibitors of heme synthesis enzymes such as itaconyl-CoA (inhibitor of ALAS (63, 153)), succinylacetone (inhibitor of PBGS/ALAD (Ebert et al., 1979)) and acifluorfen (inhibitor of PPOX (Corrigall et al., 1994; Corradi et al., 2006)) are powerful tools to probe the regulatory mechanisms of the heme synthesis pathway.

Heme synthesis is a ubiquitous process catalyzed by 8 enzymatic steps that take place in the cytosol and the mitochondria (summarized in Figure 2). The committed step of heme synthesis is the synthesis of 5-aminolevulinate (ALA) from glycine and succinyl-CoA. This process is catalyzed by ALA synthase (ALAS)/Hem1 in yeast which is localized in the mitochondrial matrix (Ajioka et al., 2006; Hunter and Ferreira, 2011) and requires the import of glycine into the mitochondria. ALA is exported from the mitochondria into the cytosol where it is converted to the monopyrrole porphobilinogen (PBG), *via* the enzyme porphobilinogen synthase (PBGS) or aminolevulinate dehydratase (ALAD). The enzyme hydroxymethylbilane synthase (HMBS) forms a linear tetrapyrrole hydrodroxymethylbilane (HMB) from four molecules of PBG. This linear tetrapyrrole is then converted to the cyclic tetrapyrrole uroporphyrinogen III *via* the enzyme uroporphyrinogen synthase (UROS). In the final cytoplasmic step of the process, uroporphyrinogen decarboxylase (UROD) catalyzes the decarboxylation of uroporphyrinogen III (UROgenIII) to synthesize coproporphyrinogen III (CPgenIII).

The terminal steps of the heme synthesis pathway occur in the mitochondria. CPgenIII is transported into the intermembrane space where it is oxidized to protoporphyrinogen IX (PPgenIX) *via* the mitochondrial inner membrane enzyme coproporphyrinogen III oxidase (CPOX) (Hamza and Dailey, 2012). PPgenIX is then transported into the mitochondrial matrix *via* a process that requires the transmembrane protein TMEM14C (Yien et al., 2014) and oxidized to form protoporphyrin IX (PPIX) *via* the matrix-facing inner membrane enzyme by protoporphyrinogen oxidase (PPOX). Finally, ferrochelatase (FECH) catalyzes the insertion of ferrous iron into the PPIX macrocycle to form heme (Hamza and Dailey, 2012).

The enzymes of the heme synthesis pathway are identical in all cells (with the exception that ALAS2 is preferentially used in erythroid heme synthesis, while ALAS1 is used in all other cells) (Dailey et al., 2005), while cells have their context-specific metabolic requirements. This opens several key questions: How do different cell types couple heme synthesis to cellular requirements? How is heme synthesis regulated in response to developmental, environmental or metabolic stimuli? While the transcriptional and translational regulation of heme synthesis genes is fairly well characterized (Drissen et al., 2005; Yu et al., 2009; Tallack et al., 2010; Wong et al., 2011; Wilkinson and Pantopoulos, 2014; Yien et al., 2014; Magor et al., 2015; Tanimura et al., 2018; Yien et al., 2018; Doty et al., 2019), the regulation of heme synthesis by the structural and functional interactions with mitochondrial homeostasis proteins is less well understood. This is a key gap in our understanding of heme metabolism because mutations in ubiquitously expressed mitochondrial homeostasis genes can cause heme defects in specific tissues (Hildick-Smith et al., 2013; Kondo et al., 2016; Piel et al., 2016; Sun et al., 2017; Kory et al., 2018; Galmozzi et al., 2019; Paul et al., 2019; Acoba et al., 2021; Bao et al., 2021; McGuire et al., 2021). This indicates that these regulatory interactions play a role in tissue-specific heme regulation. Further, elucidating the role of interactions between mitochondrial homeostasis proteins and the heme metabolon is key to understanding how metabolic stimuli can regulate heme synthesis (Medlock et al., 2015). This article will review how heme synthesis enzymes are regulated by housekeeping mitochondrial proteins, and how heme synthesis is regulated by the expression of solute carriers that enable the transport of heme intermediates and other nutrients and metabolites. Further, we will explore how mitochondrial proteins regulate mitochondrial iron import and utilization, and how mitochondrial metabolism is structurally and functionally coupled with heme synthesis. The goal of this review is to offer insights as to how these interactions have furthered our understanding of heme synthesis, and to suggest some key areas of exploration in this area.

## 3 Regulation of Mitochondrial Heme Synthesis Enzymes

### 3.1 ALAS

**FIGURE 3:**
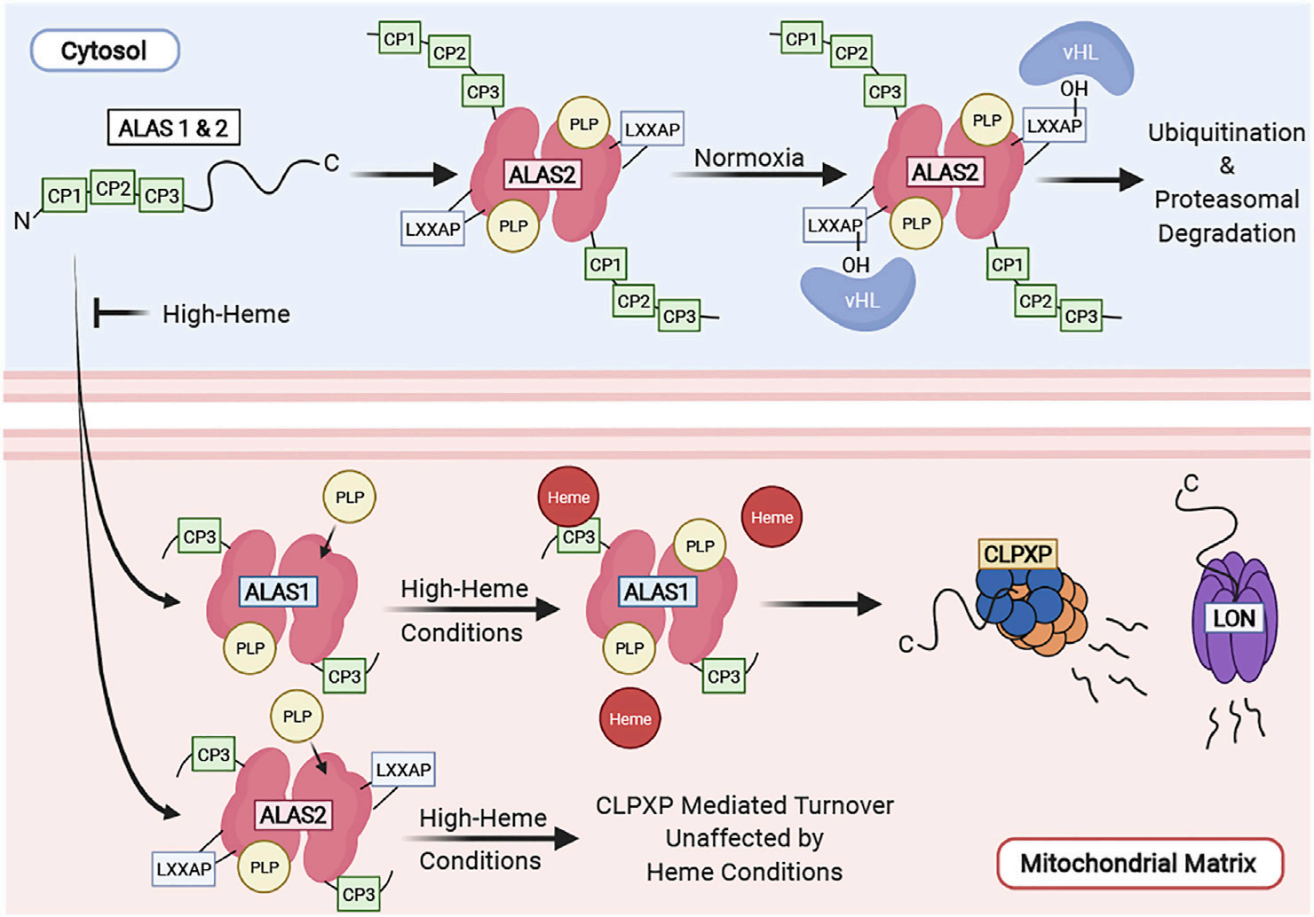
Regulation of ALAS in the cell. ALAS translocation to the mitochondrial matrix is inhibited by high heme conditions and functions as a mechanism of feedback inhibition. ALAS2 turnover is regulated by the ubiquitin-proteasome system in the cytosol and by CLPXP in the mitochondria. ALAS1 is tightly regulated by mitochondrial heme levels. High heme concentrations facilitate its degradation by the CLPXP proteolytic complex and Lon in the mitochondria.

ALAS is the rate-limiting enzyme of the heme synthesis pathway and is tightly regulated at several levels. Vertebrates possess two ALAS isozymes, ALAS 1 and 2. ALAS1 is ubiquitously expressed in all cell types, and is tightly regulated by heme (Kolluri et al., 2005), while ALAS2 is preferentially expressed in differentiating erythroid cells and is transcriptionally and translationally regulated by iron levels and erythroid differentiation (Peoc’h et al., 2019; Kramer et al., 2000; Surinya et al., 1997). Lastly, heme regulates post-translational proteolytic cleavage of ALAS and its translocation of into the mitochondria (Yamamoto et al., 1982; Lathrop and Timko, 1993; Tian et al., 2011). We have summarized the complex regulation of ALAS in Figure 3.

#### 3.1.1 Regulation of ALAS Protein Localization and Stability

ALAS2 contains an LXXLAP motif which is found in proteins that are reguated by oxygen tension. Its stability within the cytosol is regulated by oxygen levels the *via* the ubiquitin-proteasome machinery. Under normoxic conditions, the proline on the LXXAP motif of ALAS2 is hydroxylated and interacts with the von Hippel-Lindau (vHL) protein and is ubiquitinated. ALAS2 protein is also stabilized during hypoxia. In contrast, ALAS1 does not contain an LXXLAP motif and is unlikely to be regulated by oxygen tension (Abu-Farha et al., 2005).

Both ALAS1 and ALAS2 proteins contain a heme regulatory motif (HRM) consisting of three consecutive CP motifs (CP 1-3) at their N-termini. These CP motifs are so-called because they contain a dipeptide motif consisting of cysteine and proline residues (Ogawa et al., 2001). This region is required for heme binding, and heme-dependent inhibition of ALAS import into the mitochondria (Yamauchi et al., 1980; Hayashi et al., 1983; Srivastava et al., 1983; Lathrop and Timko, 1993). The CP 1 and 2 motifs of the HRM are part of the ALAS precursor protein and are cleaved upon translocation into the mitochondria. The CP3 motif remains part of the mature ALAS protein after mitochondrial translocation and is required for heme-dependent ALAS1 proteolysis by mediating its interaction with the CLPXP and LONP1 proteases (Kubota et al., 2016). In these cases, heme-dependent regulation functions as a feedback mechanism to inhibit the synthesis and translocation of ALAS to the mitochondria when free heme is in excess.

Vertebrate ALAS mitochondrial protein stability is tightly regulated by the mitochondrial AAA+ (ATPases associated with diverse cellular activities) proteases LON and CLPXP (Tian et al., 2011; Kubota et al., 2016; Rondelli et al., 2021). ALAS1 is proteolytically degraded by the Lon or CLPXP proteases in a heme dependent manner (Tian et al., 2011; Kubota et al., 2016) where addition of hemin to human hepatocytes and cell lines decreases ALAS1 protein expression *via* Lon and CLPXP, mediated by their interaction on the ALAS1 CP3 motif. This functions as a mechanism for feedback inhibition of heme synthesis. The stability of ALAS2, highly expressed in differentiating erythroid cells under the control of erythroid-specific promoters, is regulated by CLPXP. However, the ALAS2 protein is stable during erythroid differentiation, indicating that it is not degraded under high heme conditions (Rondelli et al., 2021). One caveat of these studies is hemin treatment introduces a large quantity of exogenous free heme to cells. Under physiologic conditions, the amount of free heme in cells (including in red cells, where most heme is hemoglobin bound) is very low. Hence, further studies are required to determine how heme, as bound to its physiological carriers and hemoproteins, regulates ALAS protein stability and enzyme activity *in vivo*.

The proteolytic regulation of yeast ALAS differs from that of vertebrates as yeast do not possess a CLPP gene (Kardon et al., 2015). Hence, yeast ALAS is not proteolytically regulated by the CLPXP protease. Regulation of yeast ALAS by the LON protease and heme-dependent regulation of its protein stability remains an open question.

Regulation of ALAS stability by housekeeping proteins is an increasingly important theme in understanding disorders of heme metabolism. Recently, we showed that aberrant ALAS stabilization caused by a CLPX mutation caused overproduction of ALA, leading to accumulation of PPIX, resulting in erythropoietic protoporphyria (Yien et al., 2017).

#### 3.1.2 Regulation of Cofactor Incorporation

ALAS enzymes are dependent on the cofactor pyridoxal phosphate (PLP) for their activity. Yeast ALAS/Hem1 is a homodimeric enzyme in which the one of the monomers are covalently bonded to PLP, while the other monomer is PLP free (Brown et al., 2018). In contrast, human ALAS2 is a homodimer in which both monomers are bound to PLP (Bailey et al., 2020). PLP attachment causes structural changes allowing the ALAS active site to bind substrate and is a key component of ALAS function. These data suggest that suggesting that the structural aspects of PLP incorporation are not evolutionarily conserved and can be a mode of ALAS regulation. PLP is covalently attached to an active site lysine and interacts with several other residues near the active site suggesting that PLP incorporation is a potential component of ALAS regulation (Gong et al., 1996; Astner et al., 2005; Brown et al., 2018). Indeed, the importance of PLP binding is underscored by hematologic disorders caused by mutations that disrupt PLP binding in ALAS (Cotter et al., 1995; Prades et al., 1995; Furuyama et al., 1997).

Yeast apo-ALAS requires CLPX to accelerate its incorporation of PLP (Kardon et al., 2015; Kardon et al., 2020). While the requirement for CLPX for PLP incorporation in vertebrates has not been formally tested, CLPX is not required for vertebrate ALAS activity and is therefore likely not required for incorporation of PLP (Rondelli et al., 2021). The functional differences in CLPX regulation between yeast and vertebrate ALAS are likely due to primary sequence differences as regions that are important for the interaction between yeast CLPX and ALAS are not conserved in the vertebrate ALAS proteins (Rondelli et al., 2021). Evolution of these regions may have played a role in alterations of PLP binding and its regulation for ALAS to play specific roles in adaptation of heme synthesis to cell-specific contexts. As PLP is generally maintained at a low concentration in the cell (Hamfelt, 1967; Cheung et al., 2003), and its spontaneous conjugation to apoALAS is slow (Kardon et al. 2015), it is likely that vertebrate ALAS requires other, yet to be discovered chaperones that facilitate PLP incorporation to form the holoenzyme.

In yeast, Mtm1 (originally identified as the manganese trafficking factor for mitochondrial Sod2) was identified as a potential mitochondrial transporter for PLP. Mtm1 tightly binds to PLP and is required for incorporation of PLP into mitochondrial proteins. In the absence of Mtm1, there was a significant decrease in the activity of Hem1/ALAS. While a PLP transporter should bind to PLP, the high affinity for Mtm1 to PLP may indicate that other proteins are required as carriers to transfer PLP from Mtm1 to its target proteins (Whittaker et al., 2015). Vertebrate SLC25A39, which is required for hemoglobinization and the early stages of heme synthesis, can genetically complement the iron deficiency defect in Mtm1 deficient yeast (Nilsson et al., 2009). However, the PLP binding and transport function of SLC25A39 has not been directly tested. SLC25A39 was recently implicated in the regulation of mitochondrial glutathione import in mammalian cells (Wang et al., 2021), opening the possibility that it can serve as a regulatory node for several metabolic pathways. Studies on the regulation of ALAS are summarized in Figure 3.

### 3.2 CPOX

The CPOX enzyme catalyzes the oxidation of coproporphyrinogen III to protoporphyrinogen IX. It is specific for the coproporphyrinogen III isoform, rather than the coproporphyrinogen I isoform that is not used for heme synthesis. CPOX is situated in the mitochondrial intermembrane space and is loosely attached to the mitochondrial inner membrane (Grandchamp et al., 1978). The protein contains 120 amino acid long mitochondrial leader sequence that is required for targeting to the mitochondrial intermembrane space (Dailey et al., 2005). The localization of the enzyme requires its substrate, coproporphyrinogen III, synthesized in the cytosol, to be transported through the mitochondrial outer membrane to reach CPOX in the intermembrane space.

CPOX functions as a homodimer with no cofactor (Phillips et al., 2004; Lee et al., 2005). While its reaction has been extensively studied, the mechanism by which CPOX functions is still not known (Lash, 2005; Silva and Ramos, 2011).

### 3.3 PPOX

In the penultimate step of heme synthesis, PPOX catalyzes the oxidation of protoporphyrinogen IX to protoporphyrin IX within the mitochondrial matrix. PPOX functions as a homodimer with an FAD molecule noncovalently bound to one of the subunits within the dimer (Koch et al., 2004; Corradi et al., 2006; Qin et al., 2010; Qin et al., 2011). The PPOX protein is synthesized in the cytosol and translocated into the mitochondria in a process that requires a mitochondrial targeting signal (Morgan et al., 2004; von und zu Fraunberg et al., 2003). While the structure of PPOX predicts that it is an intermembrane space-facing enzyme that docks onto FECH in such a way as to allow substrates to enter from its channel into FECH (Koch et al. 2004), live-cell peroxidase based proteomic mapping showed that PPOX binds to the mitochondrial inner membrane and faces into the mitochondrial matrix (Rhee et al., 2013).

While PPOX is not a rate-limiting enzyme in the heme synthesis pathway, its expression, like all the other heme synthesis enzymes, is upregulated during erythroid differentiation (Wong et al., 2011). To keep pace with the increase in the rate of mitochondrial iron import and ALAS activity during terminal erythropoiesis, the processes necessary for activation of PPOX and the intermediate enzymes of the heme synthesis pathway must also be upregulated to keep pace with heme synthesis and to minimize the accumulation of cytotoxic heme intermediates. PPOX requires the incorporation of its reactive FAD cofactor for its activity. While the mechanism for FAD incorporation into PPOX is unknown, potential interactions between FAD synthases and apo-flavoproteins may facilitate the “chaperoning” of FAD into the apoenzyme as FAD is being synthesized (Giancaspero et al., 2015).

In vertebrate erythroid cells, full PPOX activity requires CLPX, a mitochondrial protein unfoldase, but not CLPP, the proteolytic subunit of the CLPXP protease (Rondelli et al., 2021), suggesting that the CLPX unfoldase has a protease-independent function in regulating heme synthesis. CLPX deficiency decreases PPOX protein levels and activity and may play a role in regulating PPOX protein quality and/or cofactor incorporation.

PPOX exists within a large protein complex in the mitochondrial inner membrane (the “heme metabolon”) with other heme synthesis enzymes such as ALAS and FECH, as well as TMEM14C, which plays a role in import of protoporphyrinogen IX into the mitochondrial matrix (Medlock et al., 2015). It is likely that these physical interactions, and functional interactions with proteins like CLPX, exist for PPOX to coordinate substrate processing with downstream heme synthesis. The detailed functions of these interactions remain to be explored and will be key for understanding the regulation of PPOX in the context of mitochondrial metabolism.

### 3.4 FECH

FECH catalyzes the final step of heme synthesis which is the insertion of iron into protoporphyrin IX. FECH is synthesized in the cytosol as a pre-protein and translocated to the mitochondrial matrix where it is attached to the mitochondrial inner membrane. The enzyme exists as a homodimer. Each of the monomers in metazoan FECH is attached to a [2Fe-2S] cluster (Wu et al., 2001).

The FECH enzyme is tightly regulated. Attachment of the [2Fe-2S] cluster occurs in metazoan FECH, but not in several other organisms. While [2Fe-2S] cluster attachment does not appear to play a role in catalysis (Dailey et al., 2000), optimal activity of FECH enzymes with the [2Fe-2S] cluster requires [2Fe-2S] attachment. Various lines of evidence show that [2Fe-2S] attachment contributes to iron sensing. Under iron deficient conditions, human FECH exhibits decreased activity, while *E.coli* FECH, which does not contain a [2Fe-2S] cluster, does not (Taketani et al., 2000). In iron-deficient erythroleukemia cells, which are deficient in [Fe-S] clusters, newly synthesized apo-FECH is destabilized. This suggests that [Fe-S] cluster incorporation into FECH during protein synthesis is important for maintenance of its protein stability and maturation (Crooks et al., 2010). Decrease in FECH protein during glutaredoxin 5 (GLRX5) deficiency, which is thought to incorporate the [2Fe-2S] cluster into FECH, was observed in primary hematopoietic cells (Ye et al., 2010; Daher et al., 2019; Ducamp and Fleming, 2019), strengthening the argument that [2Fe-2S] incorporation plays a key role in maintenance of FECH protein stability. A second role of the [2Fe-2S] cluster in FECH is thought to be in mitochondrial pH sensing. In erythroid cells, dysregulation of pH by genetic disruption of the mitochondrial ATPase inhibitory factor 1 (*Atpif1*) or pharmacological inhibition of respiration causes disruption of FECH activity. A yeast FECH ortholog that does not possess a [2Fe-2S] cluster was not similarly disrupted; indeed expression of yeast FECH was able to genetically rescue the anemia in zebrafish *atpif1* mutants, suggesting the absence of a [2Fe-2S] cluster rendered yeast FECH impervious to regulation by pH. This mode of regulation did not occur in non-erythroid cells. Although knockdown of *Atpif1* in mammalian cell erythroid cell lines caused a hemoglobinization defect (Shah et al., 2012), ablation of the *Atpif1* gene in mice did not cause an overt phenotype, and the mice are normal (Nakamura et al., 2013). Hence, regulation of heme synthesis by Atpif1 is likely to be tissue specific and not conserved across species.

**FIGURE 4:**
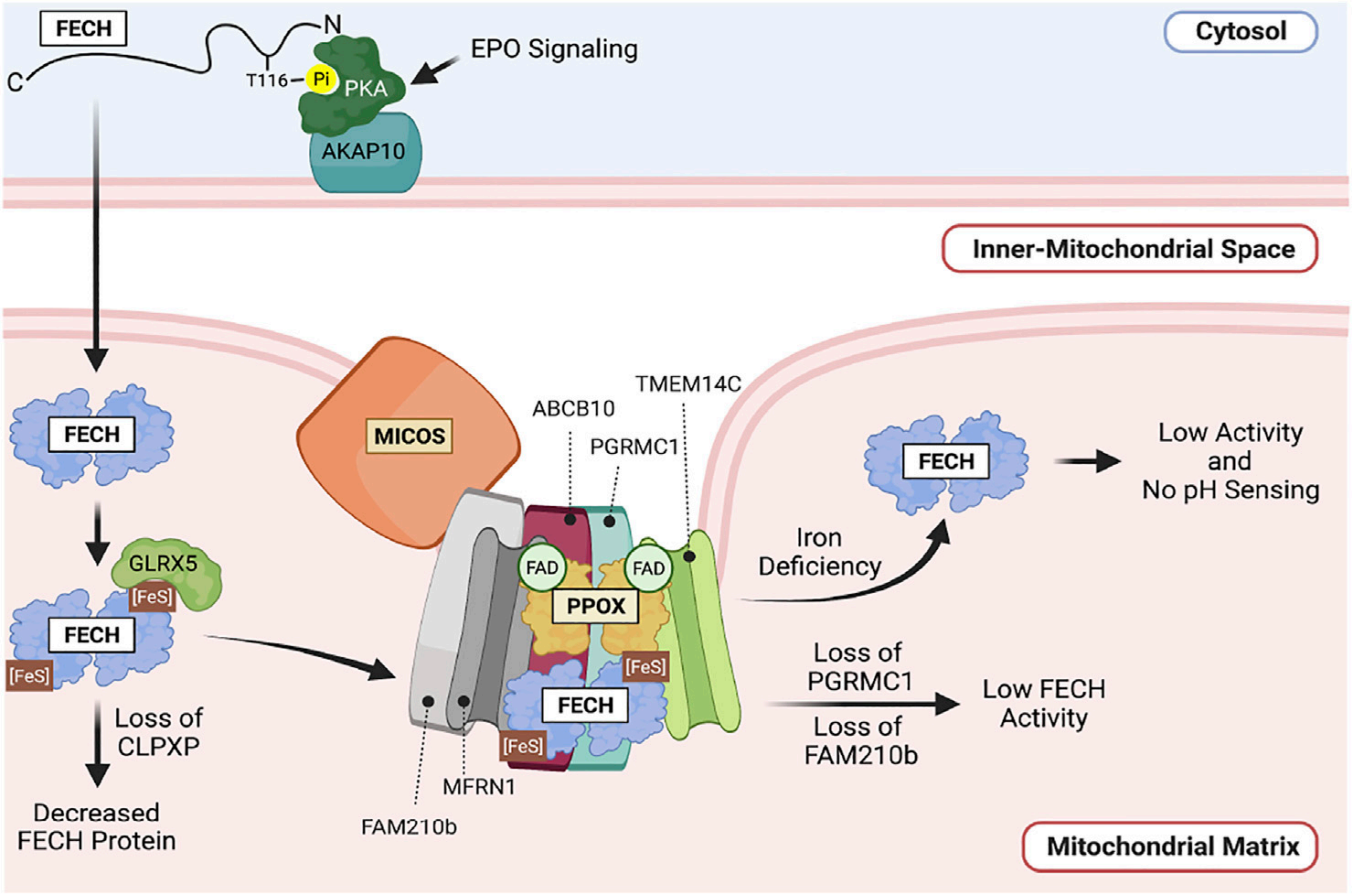
Regulation of FECH in the mitochondria. FECH is tightly regulated by post-translational modifications and Fe-S binding. FECH is phosphorylated on the outer mitochondrial membrane. This is regulated by EPO signaling. Within the mitochondrial matrix, FECH activity is regulated by pH, Fe-S binding, the CLPXP complex as well as binding to other proteins in a macromolecular complex such as MFRN1, PPOX, the MICOS complex and FAM210B.

In erythroid cells, EPO signaling during differentiation triggers the phosphorylation of FECH, which increases its activity. This occurs through the activation of PKA signaling components which are recruited to the mitochondrial outer membrane by the anchoring protein AKAP10. EPO signaling activates PKA, which in turn phosphorylates FECH, increasing its maximal activity. PKA can directly phosphorylate FECH. The presence of PKA on the mitochondrial outer membrane and mature FECH in the matrix suggests that FECH may be phosphorylated concomitant with its transport into the mitochondrial matrix (Chung et al., 2017) (Figure 4).

Optimal FECH activity requires the presence of the mitochondrial matrix localized CLPX, CLPP, and inner membrane bound FAM210B proteins. Ablation of the *Clpx* or *Clpp* genes decreased the levels of FECH protein and enzymatic activity in differentiating erythroid cells. Since the function of the CLPXP protease is to degrade proteins, yet CLPXP deficiency causes a *decrease* in FECH protein levels, it is unlikely that FECH stability is directly affected by the CLPXP protease. Rather, we propose that CLPXP may play a role in facilitating FECH protein folding, or its interactions with other proteins that are required for its maximal activity (Rondelli et al., 2021). The presence of FAM210B, a protein that is upregulated by during erythroid differentiation, is also required for maximal FECH activity in differentiating erythroid cells, even though it is not required for maintenance of FECH protein levels (Yien et al., 2018).

Importantly, regulatory mechanisms of FECH activity are integrated in the context of its integration in the heme metabolon (Medlock et al., 2015). At the most basic level, FECH interacts with PPOX. The interaction of these two enzymes is proposed to facilitate the transfer of PPIX to FECH for heme synthesis to protect PPIX from light and exposure to the aqueous environment of the cell (Proulx et al., 1993; Hamza and Dailey, 2012). FECH is also in complex with MFRN1 and ABCB10, facilitating the transfer of ferrous iron to FECH for heme synthesis (Chen et al., 2009; Chen et al., 2010). While FAM210B is necessary for the optimal activity of soluble FECH, the regulation of FECH by FAM210B is even more profound when the mitochondrial membranes are intact, as FAM210B interacts with PPOX and FECH, and is necessary for maximal import of iron into the mitochondria during erythroid differentiation and is proposed to facilitate the interaction between MFRN1 and FECH (Yien et al., 2018). Protein-protein interactions may also decrease FECH activity—interaction with PGRMC1 inhibits FECH activity, possibly by inhibiting heme release (Piel et al., 2016). The importance of mitochondrial morphology and the integrity of the mitochondrial membrane for FECH regulation is emphasized by the requirement for FECH to interact with the mitochondrial contact site and cristae organizing system (MICOS) machinery for interaction its protein partners and maximal enzymatic activity (Dietz et al., 2021). These studies emphasize the importance of studying FECH regulation within the context of its protein-protein interactions. These studies are summarized in Figure 4.

## 4 Mitochondrial Transport in Heme Synthesis

As heme synthesis occurs in the mitochondria and cytosol, the pathway requires the transport of solutes through the mitochondrial membranes. Heme and its tetrapyrrole intermediates can intercalate into lipid bilayers; as photoactive compounds, they cancatalyze the production of reactive oxygen species on exposure to light, causing tissue damage that is commonly associated with the porphyrias (Balla et al., 1993; Jeney et al., 2002). In addition, iron and tetrapyrroles are only sparingly soluble in water at physiological pH. These characteristics necessitate specialized mechanisms to facilitate the delivery of heme, its precursors and intermediates, and iron to specific cellular destinations, such as heme synthesis enzyme complexes in the mitochondrial membrane. Expression of solute transporters also adds an additional layer of control over heme synthesis that may be coordinated with metabolic requirements of the cell. Models for transport of heme synthetic intermediates have been elegantly described by Hamza and Dailey (Hamza and Dailey, 2012). Here, we provide an update on candidate transporters that were identified in the intervening years.

### 4.1 Heme Synthesis

#### 4.1.1 Glycine

The first step and committed step of heme synthesis is the condensation of glycine and succinyl-coA to form ALA. This reaction is catalyzed by the ALAS enzymes, which are localized in the mitochondrial matrix, requiring the import of glycine into the mitochondria. Glycine import is especially important in red cells, as ALAS2, the predominant ALAS isoform in erythroid cells, has a very high K_m_ (9.3 ± 1.2 mM), requiring adequate levels of glycine in the mitochondrial matrix for heme synthesis. The requisite glycine concentrations are achieved by the activity of glycine transporters in mitochondria. In vertebrates, SLC25A38, a mitochondrial protein which is highly expressed in erythroid cells carries out this function. The function of SLC25A38 as a mitochondrial glycine transporter is highly conserved across species, as is its requirement for initiating heme synthesis (Guernsey et al., 2009; Fernández-Murray et al., 2016; Lunetti et al., 2016). Its function has been demonstrated in *in vitro* glycine uptake assays with reconstituted proteoliposomes (Lunetti et al., 2016), and *in vivo*, in zebrafish mutants (Guernsey et al., 2009; Fernández-Murray et al., 2016). Homozygous loss of function mutations in the *SLC25A38* gene are a cause of transfusion-dependent congenital sideroblastic anemia (Heeney et al., 2021). Sideroblastic anemias that are caused by *SLC25A38* mutations are refractory to pyridoxine treatment, which is used to treat sideroblastic anemias caused by *ALAS2* mutations that occur in the PLP binding region (Fernández-Murray et al., 2016). While sideroblastic anemia seems to be the primary phenotypic defect associated with *SLC25A38* mutations, these patients often have other disorders. While it is not known if *SLC25A38* mutations were the primary cause of these disorders, it is likely that *SLC25A38* is also required in non-erythroid tissues, because its function is conserved in yeast (Heeney et al., 2021). In yeast, *ymc1* functions as a secondary, lower affinity glycine transporter (Fernández-Murray et al., 2016). However, a vertebrate ortholog for *ymc1* has not been identified.

#### 4.1.2 5-Aminolevulinate (ALA)

After ALA is synthesized in the mitochondrial matrix, it is exported into the cytosol where it undergoes a series of reactions catalyzed by heme synthesis enzymes. There are several candidate proteins that are important for ALA transport, but none have been directly verified *in vitro* to have ALA transport activity. ABCB10, an inner mitochondrial membrane binding, matrix facing protein (Rhee et al., 2013), plays an important role in heme synthesis in many tissues (Chen et al., 2009; Chen et al., 2010; Bayeva et al., 2013; Yamamoto et al., 2014; Seguin et al., 2017), and its activity seems to highly cell-specific. In cardiomyocytes, loss of ABCB10 decreases heme synthesis, which can be chemically complemented by ALA supplementation. Overexpression of ALAS2 or supplementation of glycine (an ALA precursor), could not rescue heme synthesis in ABCB10-deficient cardiomyocytes, leading to their conclusion the only reason for the heme defect was that ABCB10 promotes export of ALA from the mitochondria. However, the authors did not directly quantitate ALA synthesis in ABCB10-deficient cardiomyocytes, leaving open the possibility that the heme defect in these cells may also have been caused by an ALA synthesis defect (Bayeva et al., 2013). While *Abcb10* ^*−/−*^ hematopoietic cells exhibit heme defects, they accumulate PPIX, suggesting that ALA synthesis and export in these cells is likely to be normal (Yamamoto et al., 2014). This was confirmed by direct quantitation of ALA in murine erythroleukemia cells (Seguin et al., 2017). Collectively, these data suggest that if ABCB10 is required for mitochondrial export of ALA, it is only in specific cell types such as cardiomyocytes. At this point, the identity of the ALA exporter in most cell types is still an open question.

#### 4.1.3 Coproporphyrinogen III (CPgenIII)

ALA undergoes a series of enzymatic reactions in the cytosol, culminating in the synthesis of CPgenIII. At this point, CPgen must be transported across the mitochondrial outer membrane in order to reach CPOX, which is bound to the mitochondrial inner membrane, facing the intermembrane space (Grandchamp et al., 1978). The membrane transport protein, ABCB6, has been proposed as a transporter for CPgenIII. ABCB6 is an outer mitochondrial membrane protein (Paterson et al., 2007)that binds to heme, porphyrins and CPgenIII (Krishnamurthy et al., 2006; Chavan et al., 2013). Overexpression of ABCB6 in an erythroid cell line also increased heme synthesis (Krishnamurthy et al., 2006). A cryo-electron microscopy structure of human ABCB6 proposes a model by which ABCB6 may transport porphyrins (Wang et al., 2020). However, the transport mechanism proposed by Wang et al. was modeled on hemin and Fe-coproporphyrin III, which are not the physiologic substrates. Direct transport assays for CPgenIII have not been described in the literature. ABCB6 mutations are a cause of dyschromatosis universalis hereditaria (Liu et al., 2014) and ocular coloboma (Wang et al., 2012). ABCB6 is not required for erythropoiesis but rather and encodes the Langereis blood group (Helias et al., 2012), likely ruling out a major role in porphyrin transport. Genetic studies on mice suggest that plasma membrane localized ABCB6 is a porphyrin exporter, and ABCB6 mutations may modify the severity of congenital porphyrias (Fukuda et al., 2016). However, *Abcb6 −/−* mice had no overt heme synthesis phenotype, and excretion of coproporphyrins I and III, a marker of porphyrin accumulation, that indicates dysregulation of heme synthesis, did not differ from wild-type mice (Fukuda et al., 2016). Lastly, a recent study indicates that *ABCB6* mutations are not enriched in porphyria patients (Farrell et al., 2022). Collectively, these studies indicate that ABCB6 is unlikely to be the CPgenIII transporter, and the identity of the CPgenIII transporter is still unresolved.

#### 4.1.4 Protoporphyrinogen IX

Within the mitochondrial intermembrane space, CPOX oxidizes CPgenIII to PPgenIX. Hereafter, PPgenIX must be transported from the mitochondrial intermembrane space to the matrix where PPOX and FECH are present, for conversion into PPIX and heme. At this point, no direct transporter has been identified. Using biochemical assays in loss of function models, TMEM14C, a mitochondrial transmembrane protein that localizes to the inner membrane, was identified as being required for transport of PPgenIX into the mitochondrial matrix for production of PPIX by PPOX. *Tmem14c* genetrap mice die of anemia at approximately E14.5, when definitive erythropoiesis becomes required for continued embryonic development, and *Tmem14c* deficient erythroid cells exhibit developmental and metabolic defects (Yien et al., 2014). TMEM14C has three transmembrane helices that predict that it may play a transport role in the membrane (Klammt et al., 2012). However, its ability to directly transport PPgenIX across membranes has not been directly demonstrated. The requirement for TMEM14C in heme synthesis has only been demonstrated in erythroid cells, but it is likely that other proteins play analogous roles in non-erythroid cells as murine embryos can develop to E14.5, suggesting that other cell types in the developing embryo are able to synthesize heme. In primary erythroid cells, mis-splicing of *TMEM14C* is implicated in ring sideroblast formation in myelodysplastic syndrome (Clough et al., 2021).

#### 4.1.5 Transfer of PPIX From PPOX to FECH

Metabolic enzymes have long been shown to exist in macromolecular complexes that localize to specific subcellular localizations, with enzymes in the same pathway clustering together (Robinson and Srere, 1985; Campanella et al., 2005; Acín-Pérez et al., 2008). A proposed function of these large complexes is the chanelling of reaction intermediates, which is defined as the translocation of a product from one enzyme active site to another, without exposure of the reaction product to the rest of the cellular environment or bulk solution. Among the roles of these channels are to shield reactive intermediates from undergoing deleterious side reactions with cellular components, to locally concentrate reactants, and to increase the rate of association with enzymes in the pathway (Weeks et al., 2006). Such a mechanism was proposed for transport of reactants between the mitochondrially localized terminal enzymes of the heme synthesis pathway. Channeling of terminal heme synthesis reactants by complex formation between PPOX and FECH has been supported by kinetics studies (Ferreira et al., 1988). This was supported by structural biology studies which described how the membrane binding regions of PPOX can be docked onto FECH, potentially enabling a direct transfer of reactants from the active site of PPOX to that of FECH (Koch et al., 2004). TMEM14C and FECH can both be immunoprecipitated by PPOX, supporting a model in which TMEM14C, PPOX and FECH are in a complex. This would enable PPgenIX to be directly transported to PPOX *via* TMEM14C, and the resultant PPIX to be channeled to FECH (Medlock et al., 2015). This complex is predicted to be a dynamic one (Ferreira et al., 1988; Proulx et al., 1993), allowing control of heme synthesis rates *via* regulation of enzyme complex formation. The model in which PPIX is channeled to the FECH active site *via* the docking of PPOX onto FECH *via* the membrane binding region of PPOX that was proposed by Koch et al. must be reevaluated in light of the reassignment of PPOX to the mitochondrial matrix (Rhee et al., 2013). Nonetheless, the current data point to a model where heme intermediates are channeled within multi-enzyme complexes, thus allowing tight control of heme synthesis, protecting cellular compartments from deleterious side reactions with reactive heme intermediates, and maintaining high rates of heme synthesis by keeping heme intermediates localized to specific subcellular regions.

### 4.2 Heme

After heme is synthesized in the mitochondrial matrix, it must be transported to other organelles where it is used to make hemoproteins such as hemoglobin or BACH1. As heme is an iron-bound tetrapyrrole that can intercalate into membranes and catalyze the formation of reactive oxygen species, a mechanism to chaperone heme to its targets and facilitate its incorporation into hemoproteins is necessary. In erythroid cells, which synthesize most of the body’s heme, FLVCR1B, which localizes to the mitochondria, plays this role. The *Flvcr1* gene encodes two heme exporters, *Flvcr1a* and *1b*, which are alternative splice isoforms (Quigley et al., 2004; Keel et al., 2008; Chiabrando et al., 2012). *Flvcr1b* is a smaller, ubiquitously expressed splice isoform from the *Flvcr1* gene whose protein product localizes to the mitochondria. Its expression is enriched in tissues that have high rates of heme synthesis, including the bone marrow and spleen. *Flvcr1b* deficiency causes accumulation of heme in non-erythroid mitochondria and causes defects in erythroid differentiation (Chiabrando et al., 2012). Based on these data, FLVCR1B is proposed to be the mitochondrial heme exporter. Because it is required for mitochondrial heme export in HeLa cells, it is likely that its plays this role in most tissues. One caveat FLVCR1 proteins were not shown to transport or bind to heme *in vitro*. Development of *in vitro* transport assays will be critical to assess if candidates are *bona fide* heme or porphyrin transporters.

Although the most common role of heme export from the mitochondria is that of cytosolic hemoprotein synthesis because of the major role of heme in hemoglobin synthesis, heme is actually most efficiently trafficked from the mitochondrial inner membrane to the nucleus. Mitochondrial-nuclear heme trafficking was mediated by the formation of physical contacts between mitochondrial dynamics machinery (*via* proteins such as Mgm1 and Dnm1) and nuclear ER contact sites (Martinez-Guzman et al., 2020). Progesterone receptor membrane component 2 (PGRMC2) is another mitochondrial protein that is required for transport of labile heme from the mitochondria to the endoplasmic reticulum and nucleus. Here, PGRMC2 was demonstrated to facilitate the incorporation of heme into apo horseradish peroxidase (HRP), stimulating its peroxidase activity, as well as and Rev-Erbα (Galmozzi et al., 2019), providing direct *in vitro* evidence of heme chaperone activity. PGRMC2 deficiency did not cause a decrease in cytosolic heme levels, suggesting that other chaperones play the dominant role in exporting heme into the cytosol (Galmozzi et al., 2019). As PGRMC2 is most highly expressed in adipocytes, it is likely that tissue-specific mechanisms for mitochondrial heme export exist in other cell types (most prominently, erythroid cells, which may use FLVCR1B, to utilize heme for hemoglobin synthesis). Collectively, the data in these studies suggests that there are multiple mitochondrial heme transporters that serve to export heme into different subcellular compartments, and that these transport mechanisms are likely to be tissue specific.

### 4.3 Mitochondrial Iron Transport

Mitochondrial iron transport is best understood as being effected by the mitoferrin transporters, MFRN1 and MFRN2. Iron transport *via* the MFRN proteins is highly conserved from yeast to higher vertebrates, and has been demonstrated by several genetic complementation studies (Shaw et al., 2006; Froschauer et al., 2009; Paradkar et al., 2009). Most of what we know of MFRN1/2-mediated transport arises from studies of the yeast mitoferrins (Mrs3/4) or vertebrate MFRN1. Indeed, the ability of the mitoferrin proteins to transport iron was demonstrated by *in vitro* iron binding and transport assays carried out with MFRN1 (Christenson et al., 2018) or yeast Mrs3/4 (Froschauer et al., 2009). Mitoferrin proteins transport iron down a concentration gradient, and iron transport was pH dependent (Froschauer et al., 2009). Within this group, mitoferrins have some subtle differences and are tightly regulated in a cell and developmental specific manner.

The mitoferrin genes belong to a family of solute carriers named SLC25. The gene encoding MFRN1, which is enriched in sites of erythropoiesis, is *SLC25A37.* MFRN2 is ubiquitously expressed and is encoded by *SLC25A28.* These genes are orthologous to the yeast Mrs3 and Mrs4 genes. In erythroid cells, most mitochondrial iron transport is carried out by MFRN1, whose expression is upregulated during terminal erythropoiesis. *MFRN1,* the major iron transporter in erythroid cells, is transcriptionally regulated by erythroid transcription factors such as GATA1 (Yu et al., 2009; Amigo et al., 2011), and it is stabilized by the erythroid protein ABCB10, which is upregulated during terminal erythropoiesis (Chen et al., 2009; Chen et al., 2010). *MFRN2* in contrast, carries out mitochondrial iron transport in most tissues in the body, and its turnover is not regulated by ABCB10 (Paradkar et al., 2009).

Mitochondrial iron import is tightly coupled with heme synthesis. One mechanism by which this coupling occurs is through the formation of multiprotein heme synthesis complexes (Medlock et al., 2015). Biochemical studies in yeast have shown that MFRN1 interacts with FECH in a physical complex (Chen et al., 2010). In addition, maximal iron transport requires the expression of FAM210B which interacts with FECH and PPOX, and which may function as a bridging molecule to strengthen interactions between the heme synthesis enzymes to promote heme synthesis. Coupling of iron utilization, heme synthesis and mitochondrial iron transport is critical as mitochondria cannot preload iron (Lange et al., 1999).

Disruption of the *Mfrn1* and *Mfrn2* genes cause numerous defects in organismal physiology. *Mfrn1* deficiency is embryonic lethal and the embryos exhibit erythropoietic defects. *Mfrn1* deficiency in adult hematopoietic tissue causes severe anemia from erythropoietic and heme synthetic defects (Troadec et al., 2011). While *Mfrn2 ^−/−^* mice are viable, their sperm exhibit decreased motility causing decreased male fertility. Loss of both *Mfrn1* and *Mfrn2* in hepatocytes caused defects in mitochondrial iron content and physiology, leading to cell proliferation defects (Seguin et al., 2020).

While the mitoferrins appear to be the main mitochondrial iron transporters, other membrane proteins play auxiliary functions in iron transport. The Sideroflexin family members are an example of such regulatory proteins. SFXN4, an inner mitochondrial membrane protein which plays an essential role in maintenance of the oxidative phosphorylation machinery, is required for maintenance of mitochondrial iron metabolism, notably Fe-S cluster formation, and cellular iron levels (Hildick-Smith et al., 2013; Paul et al., 2019). *Sfxn4* ^*−/−*^ erythroid cells have a significantly increased percentage of iron in their mitochondria, while their cellular iron levels are decreased, showing a potential role in mitochondrial iron export. However, SFXN4 may play different roles in metabolism and various tissue types. Patients with *SFXN4* mutations suffer from mitochondriopathies which result in heterogeneous clinical manifestations including respiratory defects in the musclem, deficits in neuro-motor integration and coordination, and/or anemia (Hildick-Smith et al., 2013; Paul et al., 2019). *Sfxn2*, another member of this family, is ubiquitously expressed, particularly in the mouse kidney and liver. The SFXN2 protein localizes to the outer mitochondrial membrane, and *Sfxn2 ^−/−^* cells accumulate iron in the mitochondria while exhibiting heme synthetic defects that lead to defects in OXPHOS complex formation and function (Mon et al., 2019). Another candidate iron transporter is Rim2, a yeast mitochondrial protein that co-imports iron and pyrimidines (Froschauer et al., 2013; Knight et al., 2019). The functional orthology of Rim2 with the vertebrate proteins SLC25A33 and SLC25A36 has only been demonstrated for mitochondrial pyrimidine transport; the iron transport function of SLC25A33 and SLC25A36 has yet to be shown (Di Noia et al., 2014). Of note, deletion of any of these individual genes does not result in complete ablation of mitochondrial iron transport, suggesting that there are many redundant mechanisms for mitochondrial import, as would be expected for such an essential pathway.

Iron-sulfur (Fe-S) cluster synthesis, which depends on iron availability, is an important regulator of heme synthesis. Fe-S assembly is largely understood to occur in mitochondria, although some aspects of the pathway occur independently in the cytosol and nucleus (Rouault and Maio, 2017; Rouault, 2019; Maio and Rouault, 2020; Mühlenhoff et al., 2020). A recent study reports that the cytosolic heme synthesis enzyme, ALAD, is an Fe-S binding enzyme which requires Fe-S to maintain its enzymatic activity. This study potentially provides a mechanism by which iron homeostasis and heme synthesis are coordinated in cells (Liu et al., 2020). The importance of Fe-S assembly as a link between iron availability and heme synthesis is also underscored by the importance of frataxin, a mitochondrial protein essential for Fe-S assembly. Frataxin deficiency increases the expression of the mitoferrin proteins as well as transferrin receptor 1 (Tfr1) which mainly function to import iron, revealing a potential cause of mitochondrial iron overload (Huang et al., 2009; Crooks et al., 2014). Decrease in frataxin levels also caused downregulation of heme synthesis enzymes, iron export proteins and iron storage proteins, demonstrating its importance—and the importance of Fe-S assembly as a regulatory node by which iron availability regulates heme synthesis (Huang et al., 2009).

## 5 Metabolic Control of Heme Synthesis

It has been established that heme synthesis must be coupled with metabolic requirements to prevent toxic accumulation of heme and its intermediates. In the mitochondria, ALAS is in a multiprotein complex with succinyl-CoA synthetase (SUCLA2) which plays an essential role in the tricarboxylic acid (TCA) cycle, the main source of energy production in nucleated cells (Bishop et al., 2012; Medlock et al., 2015). The physical interaction between these two proteins was proposed to couple energy production with heme synthesis as succinyl-CoA can be fed into ALAS as a substrate. In developing erythroid cells, heme synthesis dominates mitochondrial function to meet the demand for hemoglobin. During erythropoiesis, the requirement for glycine, succinyl-CoA and iron in heme synthesis is so high that coordination with other metabolic pathways becomes essential to ensure that there are sufficient substrates for heme production as well as for ATP production and the continuation of nutrient metabolism. In developing erythroid cells, the demand for succinyl-CoA would strain on the TCA cycle if the TCA cycle were the source of succinyl-CoA in heme synthesis. A landmark study by Burch et al. demonstrated that the majority of succinyl-CoA for heme synthesis in differentiating erythroid cells was instead obtained from glutamine without passing through the TCA cycle, calling into question the biological significance of the interaction between SUCLA2 and ALAS (Burch et al., 2018).

Another important aspect of metabolic regulation is the role of iron sensing by the aconitase enzymes. Cytosolic aconitase in iron regulatory protein-1 (IRP-1) plays a central role in the iron restriction response as it is dependent on Fe-S binding to regulate the translation and stability of mRNAs that often play a key role in iron homeostasis (reviewed here (Rouault and Maio, 2017). Mitochondrial aconitase is an enzyme in the TCA cycle which is also dependent on Fe-S binding. In erythroid cells, it integrates iron sufficiency with downstream enzymes in the heme synthesis pathway, ATP production and EPO-dependent PKC signaling to erythroid developmental pathways. Mitochondrial aconitase signaling may therefore be a mechanism by which mitochondrial iron status integrates with erythropoiesis; this is underscored by the ability of isocitrate to bypass erythroid iron restriction to restore terminal erythropoeisis (Bullock et al., 2010; Richardson et al., 2013; Goldfarb et al., 2021).

Mitochondrial carriers may also play a role in metabolic regulation of heme synthesis. SFXN1 for instance, is a mitochondrial serine transporter which plays an important role in one-carbon metabolism (Kory et al., 2018). SFXN1 plays several roles in heme synthesis. Serine, the transport substrate of SFXN1, is converted to glycine, which is key substrate for the committed step of heme synthesis. In the absence of SFXN1, the rate of serine to glycine conversion significantly decreased, which would impair the first step of heme synthesis particularly in erythroid cells. SFXN1 was also required for maintenance of alpha ketoglutarate levels, an important source of succinyl-CoA for heme synthesis (Burch et al., 2018). Lastly, SFXN1 deficiency decreased the mRNA levels of *CPOX* and *FECH*, as well as the levels of FECH protein. Heme is deficient in *SFXN1-*deficient cells, which in turn leads to defects in the activities of OXPHOS complexes (Acoba et al., 2021).

Heme synthesis may also be inhibited by cellular metabolites, and one example of this is the competitive inhibition of ALAS2 activity by itaconate, a metabolite produced by macrophages during inflammation. Itaconate production, by inhibiting ALAS2 activity, may therefore play a role in anemia of inflammation in addition to iron restriction (Marcero et al., 2021).

Terminal erythroid differentiation is marked by the shift in reliance on oxidative phosphorylation to glycolysis for energy production. FAM210B, which is required for maximal mitochondrial iron import and FECH activity in erythroid cells, also plays a role in metabolic reprogramming. Loss of FAM210B caused glycolysis and increased mitochondrial respiration in cancer cells by reducing expression of pyruvate dehydrogenase kinase 4 (PDK4) (Sun et al., 2017). While it has not been experimentally demonstrated, it is tempting to speculate that the increase in FAM210B expression in erythroid cells during terminal differentiation may play a role in regulating its metabolic shift to glycolysis, concomitant to its role in heme and iron regulation. We have provided a table of mitochondrial housekeeping proteins with known effects on heme synthesis for quick reference (Table 1).

**TABLE 1:** Mitochondrial housekeeping proteins with known effects on heme synthesis.

Housekeeping protein	Role in heme synthesis	Citations
ABCB6	Possible, but unlikely, porphyrin transporter	(Krishnamurthy et al., 2006; Paterson et al., 2007; Helias et al., 2012; Wang et al., 2012; Chavan et al., 2013; Liu et al., 2014; Fukuda et al., 2016; Wang et al., 2020; Farrell et al., 2022)
ABCB10	Stabilizes SLC25A37 and regulates ALA export	(Chen et al., 2009; Chen et al., 2010; Bayeva et al., 2013; Yamamoto et al., 2014; Seguin et al., 2017)
Aconitase 2	Integration of iron status with erythroid development	(Bullock et al., 2010; Richardson et al., 2013; Goldfarb et al., 2021)
AKAP10	Regulates PKA mediated FECH activity	Chung et al. (2017)
ATPIF	Regulates FECH activity (?)	(Shah et al., 2012; Nakamura et al., 2013)
CLPP	ALAS turnover	(Kubota et al., 2016; Rondelli et al., 2021)
CLPX	ALAS turnover, PPOX and FECH regulation; ALAS activation in yeast	(Peoc’h et al., 2019; Phillips et al., 2019; Yien et al., 2017; Ducamp et al., 2021; Kubota et al., 2016; Rondelli et al., 2021; Kardon et al., 2015; Brown et al., 2018; Kardon et al., 2020)
FLVCR1b	Mitochondrial heme exporter	(Quigley et al., 2004; Keel et al., 2008; Chiabrando et al., 2012; Doty et al., 2019)
Lonp1	ALAS proteolysis	(Tian et al., 2011; Kubota et al., 2016)
MICOS complex	Regulates FECH activity	Dietz et al. (2021)
PGRMC1	Regulates FECH activity	Piel et al. (2016)
PGRMC2	Exports heme	Galmozzi et al. (2019)
SFXN1	FeS cluster metabolism Serine import	(Kory et al., 2018; Acoba et al., 2021; Bao et al., 2021)
SFXN2	Mitochondrial iron metabolism and regulation of OXPHOS	Mon et al. (2019)
SFXN4	Cellular respiration; iron regulation	(Hildick-Smith et al., 2013; Paul et al., 2019)
SLC25A37 (MFRN1)	Mitochondrial iron import	(Shaw et al., 2006; Christenson et al., 2018)
SLC25A38	Glycine import	(Guernsey et al., 2009; Fernández-Murray et al., 2016; Heeney et al., 2021)
SLC25A39/Mtm1	Possible mitochondrial PLP import and glutathione regulation	(Nilsson et al., 2009; Whittaker et al., 2015; Wang et al., 2021)
SLC25a33/36/Rim2	Pyrimidine and iron transporter	(Froschauer et al., 2013; Di Noia et al., 2014; Knight et al., 2019)
TMEM14c	PPgenIX import	(Nilsson et al., 2009; Yien et al., 2014; Clough et al., 2021)

## 6 Conclusion

We envision that the field of heme regulation and mitochondrial homeostasis will continue to grow as technical advances enable us to address the gaps in our knowledge. Advances in imaging, cell biology, genetic engineering, and mitochondrial physiology have enabled us to experimentally address questions that would have been unimaginable just 20 years ago. Because initial studies of heme metabolism and regulation in erythroid cells gave rise to techniques that allowed us to interrogate heme metabolism in different tissue types, we now have a far greater understanding of how metabolic pathways integrate with and respond to the extracellular environment, metabolic requirements, and developmental stimuli. Because heme synthesis is so tightly regulated and tied to specific cellular requirements, we predict that studies of heme regulation will provide a paradigm with which to study how nutrient metabolism in general is regulated in individual cell types.
